# Outcome of peri‐implant maintenance care in patients with an implant‐supported lower denture—A 3.5‐year retrospective analysis

**DOI:** 10.1111/cid.12963

**Published:** 2021-01-18

**Authors:** Pieter Leo van der Moolen, Ben Jeroen Post, Dagmar Else Slot, Fridus August van der Weijden

**Affiliations:** ^1^ Department of Periodontology, Academic Centre for Dentistry Amsterdam (ACTA) University of Amsterdam and Vrije Universiteit Amsterdam Amsterdam The Netherlands; ^2^ Clinic for Implantology Utrecht Utrecht The Netherlands

**Keywords:** bleeding on probing, edentulous mandible, implant‐supported overdenture, non compliance, peri‐implant tissues, pocket probing depth, supportive care

## Abstract

**Background:**

Implant‐supported overdentures represent a successful treatment for edentulous patients. As early diagnosis, detection and supportive care are considered key factors for the prevention of peri‐implant diseases, consistent maintenance of these implants is becoming increasingly relevant.

**Purpose:**

This retrospective analysis evaluated a cohort of edentulous patients with a mandibular implant‐supported overdenture over a period of 3.5 years during which the peri‐implant tissues were assessed.

**Materials and Methods:**

A total of 108 patients that had consistently adhered to the annual maintenance appointments was selected. The clinical peri‐implant pocket probing depth (PiPPD) and peri‐implant bleeding on probing score (PiBOP) were investigated. Data from the 3.5‐year follow‐up were compared to data from the baseline assessment.

**Results:**

A 100% implant survival was reported after 3.5 years. The mean PiBOP showed a significant decrease over time (*P* = .028). The mean PiPPD was found significantly deeper for male patients both at baseline (*P* = .004) and 3.5‐year follow‐up (*P* < .001). Besides, the PiPPD for locator anchorages was found significantly deeper compared to ball anchorages at the 3.5‐year follow‐up (*P* = .026).

**Conclusion:**

In those patients that adhered to the annual maintenance visits during the 3.5 years after implant surgery a stable peri‐implant condition was observed. As future consideration, the comparison of the clinical outcomes of patients participating in the maintenance program with those that did not would make this observation even more meaningful.

**What is known**:

- The long‐term success of dental implants is well established, as is the treatment of implant‐supported lower dentures.
- Recently, there has been a growing interest in evaluating the supportive care of dental implants in order to prevent implant failure due to peri‐implant mucositis and peri‐implantitis and to maintain long‐term implant success rates.

**What this study adds**:

- This retrospective analysis contributes to the understanding of peri‐implant attachment loss, the success of locator and ball attachment systems and preventive measures in edentulous patients with an implant‐supported lower denture.

## 1 INTRODUCTION

Implant‐supported overdentures represent an economical, feasible, and highly successful treatment for edentulous patients.^1^, ^2^ In 2002, the McGill Consensus Statement on Overdentures defined two‐implant overdentures in the edentulous mandible as the minimum standard of care.^3^

Over the past decades, treatment with dental implants of different designs has become a predictable treatment approach with limited complications.^4^ Long‐term evaluations representing an observation period of 20 years have shown that a cumulative implant survival rate over 90% can be achieved in case of two‐implant lower dentures.^5^ Moreover, after 24 years, still a cumulative implant survival rate of 85% can be achieved.^6^ In order to obtain long‐term success of the dental implants, it is crucial to prevent, diagnose, and treat peri‐implant diseases at an early stage.^7^ Basically, this can be achieved by an appropriate level of oral hygiene. However, not all patients are able to achieve a sufficient level of self‐care and therefore regular professional re‐evaluation and supportive care is mandatory.

Several clinical parameters are available to evaluate the health of the peri‐implant tissues. Visual inspection and palpation should be used as clinical methods to detect the presence of an inflammation.^8^ In addition, peri‐implant pocket probing depth (PiPPD) has been proposed as an important diagnostic tool.^9^ Studies with experimental peri‐implantitis showed a relation between an increase in pocket probing depth, bone loss and clinical attachment around dental implants.^10^, ^11^ PiPPD measurements over time are useful to assess whether a significant change (≥ 2 mm increase) from the baseline assessment occurs.^12^ Consequently, the baseline assessment provides essential information.

Long‐term examinations in humans have shown that a healthy implant sulcus does not always resemble a probing pocket depth ≤ 3 mm, but can vary between 4 and 6 mm.^13^ Besides, the clinical probing depth can be misguided by several factors such as probing force, level of placement/bone, implant type, or construction mucosa.^10^ So, it is not possible to define a range of probing depths for dental implants compatible with health. Therefore, the pocket probing depth measurement should not be used on its own to diagnose a disease. This diagnostic tool should be related to a baseline measurement following the placement of the supra‐structure and be combined with other clinical symptoms such as the peri‐implant bleeding on probing score (PiBOP), evaluation of bone loss on radiographs and the presence of suppuration.^10^ Bleeding of the peri‐implant soft tissues upon gentle probing (PiBOP) is assumed as an efficient parameter for the diagnosis of mucosal inflammation and monitoring of the mucosal tissue around dental implants.^14^, ^15^ Alongside the patient's oral hygiene, other (risk‐)factors like age and gender may have an impact on the peri‐implant condition.

This retrospective analysis evaluated a cohort of edentulous patients with an implant‐supported lower denture over a period of 3.5 years who had consistently adhered to the yearly maintenance appointments during which the health of the peri‐implant tissues was assessed and elective supportive care was provided.

## 2 MATERIALS AND METHODS

This retrospective analysis was prepared according to the guidelines suggested by the STROBE checklist. This checklist recommends items that should be included in reports of observational studies and studies using routinely collected observational data.

This research report was approved by the Institutional Review Board of the Academic Centre for Dentistry Amsterdam (METc‐VUmc). The provided protocol‐number for this report is “2018070.”

### 2.1 Study population

All patients selected for this retrospective analysis had received a two‐implant overdenture in the mandible in a private clinic restricted to implantology in Utrecht, the Netherlands, between January 2011 and December 2015. Indication for an implant‐supported lower denture was the following:

- Fully edentulous
- Insufficient retention of a conventional mandibular denture with associated problems
- Sufficient residual bone height (≥ 8 mm) for implant placement between the mental foramina, as assessed via a lateral radiograph.

The study population consisted of patients in good general health and included those with well‐controlled diabetes mellitus and those using anticoagulants. Subjects with a history of cranial radiotherapy or those using more than 3 years bisphosphonate medication were excluded.

The patients received two tissue‐level Straumann Standard implants (Institut Straumann AG, Basel, Switzerland) for the implant‐supported lower denture. The implants were located approximately at the 33 to 32 and 42 to 43 locations. Implant diameters varied between 3.3 and 4.1 mm based on the choice of the operator in relation to the width of the remaining alveolar bone process.

Retrospective data were collected of those patients that had attended their annual maintenance visits during a period of at least 3.5 years. Those who failed to comply to the annual maintenance visits and those with missing data were excluded.

### 2.2 Surgical and prosthetic procedure

In short, the implant surgery procedure was performed by an experienced operator, accredited as such by the Dutch Association for Oral Implantology (NVOI). Perioperative antimicrobial prophylaxis was provided during 5 days, starting 2 days before the implant surgery up to 3 days afterward (amoxicillin, 375 mg, three times a day). A mid‐crestal incision with a midline vertical releasing incision was used for flap elevation. The site of the implant was prepared according to the manufacturer's protocol. Irrespective of implant length, the screw threads were entirely embedded in the mandibular bone. A prefabricated surgical reference guide was used during the implant procedure. After placement, the implants were covered with Regular Neck healing abutments (2‐4 mm height) in order to provide proper gingival transformation.^16^

The lower denture was supported by either a locator (Zest Anchors LLC., Escondido, California) or ball (Straumann Retentive Anchor H3, 4 mm, Ti; REF 048.439) anchorage system at the indication of the prosthodontist responsible for the treatment of the implant‐supported restoration.

### 2.3 Maintenance care

For maintenance care of the dental implants and their surrounding tissues, a guideline is available from the Dutch Association for Periodontology (NVvP) in collaboration with the NVOI.^17^ According to this guideline approximately 6 to 8 weeks, but at least within 6 months, after placement of the final restoration the patients should be recalled for the first clinical and radiographic evaluation; the baseline assessment. Patients received another appointment 6 months later to evaluate their level of self‐care and subsequently they were recalled every year for re‐evaluation of the peri‐implant condition.

During the baseline visit and annual maintenance visits the health of the peri‐implant tissues was clinically investigated. The pocket probing depth (PiPPD) and bleeding tendency (PiBOP) were assessed using a pressure‐sensitive probe (Click‐Probe, Kerr Hawe Neos scale: 3‐5 to 7‐10 mm). PiPPD‐measurements were rounded off to the nearest millimeter at six sites of each implant: disto‐vestibular (DV), vestibular (V), mesio‐vestibular (MV), disto‐lingual (DL), lingual (L), and mesio‐lingual (ML). The mean PiBOP was calculated as a percentage of these sites.

At the annual maintenance visits, any visible plaque and calculus were removed manually with carbon fiber instruments and/or an air‐polisher (Air‐Flow Handy 3.0, EMS – Electro Medical Systems SA, Nyon, Switzerland). If needed, oral hygiene instructions for appropriate self‐care were given including instructions for denture hygiene purposes. Patients were advised to use an electric toothbrush (Braun/Oral‐B Vitality, Procter & Gamble, Rotterdam, The Netherlands).

During each maintenance visit the oral soft tissues were assessed as well. If a patient suffered from an *oropharyngeal candidiasis*, an antimycotic treatment of either Miconazole (Daktarin, 2.5‐5 mL, four times a day) or Nystatin (Nystatin Labaz, 4‐6 mL, four times a day) was prescribed. Miconazole is considered the first choice antimycotic treatment for *oropharyngeal candidiasis* and Nystatin was applied only if Miconazole was contraindicated.^18^

### 2.4 Statistical analysis

All data were entered into an Excel file at the Clinic for Implantology Utrecht. In order to secure patients' privacy, further analysis was performed with an anonymous data set. The mean values of the different variables were analyzed for each implant and patient. The patient was used as the statistical unit. In order to analyze whether there was a change from baseline in the health of the peri‐implant tissues over time, the mean PiPPD and PiBOP were compared to these variables at the 3.5‐year follow‐up.

For those patients that fulfilled the 3.5 years of maintenance care a Paired‐samples T‐test was used to analyze the mean differences over time and an Independent‐samples T‐test was used for performing sub‐analysis comparing sub‐groups at one point in time. It was a priori decided to perform a sub‐analysis on implant location, gender and anchorage system. The level of significance was set at *P* ≤ .05. All tests were performed using a statistical software package (SPSS Statistics 26, IBM, Chicago, Illinois).

## 3 RESULTS

During the reviewed period between January 2011 and December 2015, 232 patients had received a two‐implant overdenture in the mandible. A total number of 108 patients met the inclusion criteria and had complied with their annual maintenance visits for a period of 3.5 years (Table 1).

**TABLE 1:** Number of included and excluded patients and reasons for exclusion

Study population	
Total number of patients treated between January 2011 and December 2015	232
Number of included patients	108
Number of excluded patients	124
**Reason for exclusion**	
Absence during maintenance care	102
Missing data^a^	22

a Missing data were referred to as incomplete or missing evaluations on annual maintenance visits within the 3.5 years of maintenance care while the subject fulfilled the 3.5‐year period of the study.

Forty‐three percent of the patients were male and the mean age at the time of implant surgery was 66 years (range: 39‐86 years). The type of anchorage system was either a locator (66%) or ball anchorage system (34%). None of the 216 implants were lost during the maintenance care of 3.5 years. Other prosthetic complications were not documented. The descriptive statistics are presented in Table 2.

**TABLE 2:** Patient and anchorage system demographics

Demographics	
Mean age of surgery (SD)	66 (9.04)
Age range (in years)	39 – 86
No. of male patients	46 (43%)
No. of female patients	62 (57%)
No. of patients with locator attachments	71 (66%)
No. of patients with ball attachments	37 (34%)

### 3.1 General analysis of PiBOP and PiPPD

There was a statistically significant difference between the baseline and 3.5‐year follow‐up in the mean PiBOP (*P* = .028). The mean PiBOP of 13.56% at baseline decreased to 8.43% at the 3.5‐year assessment (−5.13). For the PiPPD the mean 2.09 mm at baseline numerically increases slightly up to a mean pocket probing depth of 2.14 mm at 3.5 years but this change was not found to be statistically significant. This outcome remains the same if data are analyzed for both implant locations separately. When comparing their mean values at baseline and 3.5 years the 32‐ and the 42‐implant show a small numerical increase on the PiPPD of 0.08 and 0.02 mm, respectively, both not statistically significant (Table 3).

**TABLE 3:** Analysis of overall mean (SD) bleeding on probing (PiBOP) and pocket probing depth (PiPPD) at baseline and 3.5‐year follow‐up

Mean (SD)				
	Baseline	3.5 years	Difference	*P‐*value^a^
PiBOP (n = 108)	13.56 (18.34)	8.43 (15.81)	–5.13	.028
PiPPD (n = 108)	2.09 (0.53)	2.14 (0.54)	0.05	.360
Implant 32	2.07 (0.57)	2.15 (0.58)	0.08	.193
Implant 42	2.11 (0.56)	2.13 (0.56)	0.02	.701

Abbreviations: PiBOP, peri‐implant bleeding on probing in %. PiPPD, peri‐implant pocket probing depth in mm.

^a^ Paired‐samples *t‐*test; between group comparison. *P* < .05.

### 3.2 Sub‐analysis by gender

A sub‐analysis by gender was performed to see if there was a difference in PiBOP and/or PiPPD between males (n = 46) and females (n = 62). For the mean PiBOP there was no statistically significant difference at baseline nor after 3.5 years of supportive care. The mean PiBOP for both represented a numerical decrease of 6.91% and 3.82%, respectively. For the PiPPD there was a statistically significant difference between male and female patients, both at baseline (*P* = .002) and 3.5‐year follow‐up (*P* < .001). The mean PiPPD at baseline differed 0.31 mm between a mean of 2.27 and 1.96 mm for men and women, respectively. The mean PiPPD at 3.5‐year assessment differed 0.39 mm between a mean of 2.36 and 1.97 mm for men and women, respectively (Table 4).

**TABLE 4:** Analysis of overall mean (SD) bleeding on probing (PiBOP) and pocket probing depth (PiPPD) by gender at baseline and 3.5‐year follow‐up

Mean (SD)						
	Baseline		3.5 years		Difference	
	PiBOP	PiPPD	PiBOP	PiPPD	PiBOP	PiPPD
Male (n = 46)	14.74 (19.49)	2.27 (0.506)	7.83 (14.69)	2.36 (0.564)	−6.91	0.09
Female (n = 62)	12.69 (17.61)	1.96 (0.510)	8.87 (16.71)	1.97 (0.462)	−3.82	0.01
*P*‐value^a^	0.570	0.002	0.736	0.000	‐	‐

Abbreviations: PiBOP, peri‐implant bleeding on probing in %. PiPPD, peri‐implant pocket probing depth in mm.

a Independent‐samples *t*‐test; within group comparison. *P* < .05.

### 3.3 Sub‐analysis by anchorage system

A sub‐analysis by anchorage system was performed between locator (n = 71) and ball (n = 37) anchors. The mean PiBOP of the anchorage systems showed no statistically significant difference at baseline and 3.5‐year follow‐up. However, for both attachments, there was found a numerical decrease in the mean PiBOP. Also for the mean PiPPD, there was no statistically significant difference between the locator and ball anchorage systems at baseline. Yet, after 3.5 years of maintenance care the PiPPD for the locator anchorage system was significantly deeper compared to the ball anchorage system (*P* = .026). These data are presented in Table 5.

**TABLE 5:** Analysis of overall mean (SD) bleeding on probing (PiBOP) and pocket probing depth (PiPPD) by anchorage system at baseline and 3.5‐year follow‐up

Mean (SD)						
	Baseline		3.5 years		Difference	
	PiBOP	PiPPD	PiBOP	PiPPD	PiBOP	PiPPD
Locator (n = 71)	13.82 (18.17)	2.06 (0.56)	8.68 (17.23)	2.22 (0.56)	−5.14	0.16
Ball (n = 37)	13.08 (19.01)	2.15 (0.48)	7.95 (12.87)	1.98 (0.48)	−5.13	−0.17
*P*‐value^a^	.845	.387	.821	.026	‐	‐

Abbreviations: PiBOP, peri‐implant bleeding on probing in %. PiPPD, peri‐implant pocket probing depth in mm.

^a^ Independent‐samples *t*‐test; within group comparison. *P* < .05.

### 3.4 Oral soft tissue assessment

During the reviewed period, the oral soft tissues were assessed as well and incidents of an *oropharyngeal candidiasis* were noted. The incidence of an infection amongst the patients represented 19 cases in total (n = 108), of which five were diagnosed at the baseline assessment and 14 did so within the 3.5 years of maintenance care. There were two patients who suffered from an *oropharyngeal candidiasis* at the 3.5‐year assessment (1.9%). The other 12 incidents were diagnosed at one of the other yearly assessments during maintenance care (Table 6).

**TABLE 6:** Oral soft tissue assessment within 3.5 years of maintenance care

Incidents	
Total incidents of an *oropharyngeal candidiasis*	19
No. of post‐surgical *oropharyngeal candidiasis* events	5 (4%)
No. of *oropharyngeal candidiasis* events within 3.5‐year assessment	14 (12.5%)
No. of patients that suffered from more than one *oropharyngeal candidiasis*	2 (1.8%)

## 4 DISCUSSION

This retrospective analysis evaluated the PiPPD and PiBOP in patients with a two‐implant overdenture in the edentulous mandible over a follow‐up period of 3.5 years. This analysis was conducted to examine the adherence to post‐surgical care and to evaluate the health of the peri‐implant tissues of patients that have consistently adhered to the annual maintenance visits and supportive care. This research report showed that approximately 50% of the patients did not adhere to the yearly maintenance appointments. For those that did adhere, a 100% implant survival rate over the reviewed period was observed.

### 4.1 PiBOP and PiPPD

In this retrospective analysis, the mean PiBOP decreased significantly over time between the baseline and the 3.5‐year assessment with 5.13%. Absence of bleeding is a good indicator of a stable peri‐implant condition.^19^ However, there are cases in which some bleeding may occur even in a stable peri‐implant environment. This PiBOP can then be caused by disruption of the epithelial junction.^20^ The findings of this report correspond to the study of Wang, Renvert and Wang (2019)^19^ where a reduction in the bleeding tendency is a clinical indication of healthier peri‐implant tissues. Moreover, patients who attend to annual appointments for maintenance purposes represent better clinical peri‐implant conditions in terms of a reduction in bleeding on probing.^21^

For the PiPPD, a healthy implant sulcus does not always resemble a pocket probing depth ≤ 3 mm, but can vary between 4 and 6 mm after long‐term examinations.^13^ In this retrospective analysis, the mean PiPPD increases slightly. Nevertheless, the clinical relevance of the observed numerical increase is questionable. Although there is a slight change in PiPPD, none of the patients showed an increase ≥ 2 mm from the baseline assessment which is considered to be a relevant sign for deterioration.^12^ Wang et al concluded that only in some cases pocket reduction occurs when evaluating the peri‐implant condition of dental implants.^19^ Therefore, a slight increase in pocket probing depth as presented in this report does not imply that the health of the peri‐implant tissues is at risk. However, notable differences in situations over time, compared to previous recordings, can be an alarming sign. If a continuing increase of the PiPPD is noticed this might be a sign of disease for which a proper treatment should be initiated before long. After that, a strict treatment protocol is crucial to guarantee and/or maintain a stable peri‐implant condition.^7^

### 4.2 Gender

Evaluation of gender differences showed only a numerical decrease on PiBOP of 6.91% for men and 3.82% for women. These differences were not statistically significant, but are in accordance with the positive effect of attendance to annual maintenance.^21^ Whereas females tend to have a higher level of bleeding on probing^22^ the results of this retrospective analysis show no distinct difference between men and women for which the collected data do not provide an explanation.

The mean PiPPD for men as compared to women was significantly deeper at both assessments. These findings do not correspond to the current literature where women tend to have a deeper pocket probing depth than men, supposedly due to hormone fluctuation.^23^ Also for this, no explanation can be given based on the current findings, but may in part be influenced by the lower number of men included in this report.

### 4.3 Survival rate

The high survival rate of the implants (100%) in this retrospective analysis could be explained by their location in the mandible. In general, the mandible has a denser and thicker cortical layer than the maxilla, especially at the inter‐foraminal region. Density and bone quality both have a great influence on the success rate of treatment with dental implants.^24^ A higher bone density is one of the most important factors related to good implant treatment outcomes.^25^ The chance of survival of small‐diameter implants is shown to be higher in the mandible as well as compared to the maxilla.^26^ Other reports conclude that there is no difference in the long‐term survival rate of implants between the mandible and maxilla.^27^ Yet, small‐diameter implants show more marginal bone loss compared to regular diameter implants in the mandible even in the first three years after implant surgery.^16^

### 4.4 Anchorage systems

Despite the long‐term success of dental implants used for implant‐supported overdentures one could wonder whether the material properties influence not only the biomechanical outcomes but the tissue results of these implants as well. A number of either two or four implants used for an overdenture have been proven to have no significant difference on the peri‐implant condition.^28^ Some controversy persists about the type of anchorage system. The system used for retention was claimed to have no effect on the soft tissues.^29^, ^30^, ^31^ However, more than 15 years later too little evidence is present to determine the effectiveness of different anchorage systems on prosthodontic maintenance, prosthodontic success, patient satisfaction, patient preference or costs.^32^ While the health of the peri‐implant tissues is not always involved in these maintenance assessments, the clinical condition of these soft tissues requires attention.

In this report either a locator or ball anchorage system was used. Analysis showed that the PiPPD on average decreases within the ball attachment group and the ball anchorage system tends to perform better. Ball attachment systems do need less active supportive care than locator attachment systems because the supra structure is more easily to brush for the patient.^33^ In contrast with the beneficial characteristics of the ball attachment system for patients, the results of this retrospective analysis could also be due to overgrowth of the peri‐implant mucosa which hinders proper PiPPD measurement at the annual maintenance visits. The origin of this peri‐abutment mucosal enlargement can go either way. As in healthy cases the overgrowth can be caused by keratinized tissues, the overgrowth in an inflamed case is caused by swelling indicating plaque accumulation.^34^ Considering the general decrease in PiBOP the latter is unlikely.

### 4.5 Oral soft tissues

In this retrospective analysis the oral soft tissues were assessed during each maintenance visit. The number of patients with an *oropharyngeal candidiasis* was limited. Furthermore, only two patients with this condition were diagnosed as such at the assessment after 3.5 years of supportive care. The other incidents were encountered at different time points during the 3.5‐year follow‐up period. This makes it hard to evaluate the impact of an *oropharyngeal candidiasis* on the peri‐implant condition, so whether an *oropharyngeal candidiasis* affects the health of the peri‐implant tissues deserves further examination.

## 5 LIMITATIONS

### 5.1 Adherence

The importance of regular maintenance care for patients with implant‐supported overdentures is well established.^35^ Of the 232 patients that were treated in this research report, 102 patients had to be excluded because they did not adhere to the designated maintenance visits (Table 1). Considering that all patients are told at intake and also in a written leaflet at the baseline assessment that adherence to maintenance visits is essential for the health of the peri‐implant tissues, it is surprising that almost fifty percent of all patients does not comply. From a study for periodontal therapy it was found that after instruction on oral hygiene less than half of the patients still used interproximal cleaning aids at the end of three years.^36^ Another study reported that two thirds of the patients who drop out of suggested oral hygiene regimes will do so within 90 days.^37^ These findings tell us that motivating patients for consistent self‐care and maintenance visits is challenging. Although not shown in the results, patients that dropped out of this report do so before or after the baseline assessment.

The absence of these drop‐outs in the analysis may have introduced a bias in the results towards a seemingly beneficial effect of the maintenance treatment. In the light of this possibility, one should interpret the findings of this retrospective analysis with some caution.^38^ The interpretation of the results would benefit from a comparison with the clinical outcomes of those patients that did not adhere to the maintenance program. Although not ideal, the results of the present cohort study are a good representation of what can be achieved in clinical practice every day.

There are several hypotheses to explain the behavior of non‐compliance. Unintentional non‐compliance may be linked to insufficient resources and intentional non‐compliance may be related to motivation.^39^ Financial barriers have shown to be one of the reasons that may keep patients away from complying to maintenance visits.^40^ The fact that in the Netherlands the surgical implant procedure is covered by regular insurance while the maintenance care is not, makes it plausible to state that this could be a major reason for non‐compliance in this research report.

Different studies have been conducted to find out which approaches can improve compliance and/or adherence. Reducing the financial barrier in terms of third‐party payments can be a solution.^41^ It also seems that careful, detailed and continuing instruction in oral hygiene followed by positive feedback and reinforcement can improve oral hygiene habits to some degree. While the frequency of recall can be debated, regular maintenance care does keep the vast majority of patients under control.^42^ When time intervals of follow‐ups are longer, patients tend to show a decrease in compliance.^43^

### 5.2 Study design

Using electronic patient data in dental research, like in this retrospective analysis, is nascent but accelerating. The new possibilities and opportunities that come with digital EDR/EPR/EHR files are substantial. On the other hand, reusing and depending on previously documented data has its limitations. Once the data is documented as it is, one is restricted to the information that is captured. Besides this inability to capture study‐specific data, the quality of the practice based data is always less as compared to data collected according to a prospective research design.^44^ The referral practice operated according to a strict treatment and documentation protocol. Still, 22 cases of the 232 patients treated in the 5‐year period were either incomplete or reported missing data and were therefore excluded from the analysis.

### 5.3 Future research

An increasing life expectancy combined with improved oral health (in the Netherlands) is plausible to lead to less denture wearers in the future. Yet, those individuals that already have an overdenture have to account for a longer edentulous period. For the latter, annual maintenance assessments and re‐evaluation are necessary to maintain a stable peri‐implant condition. Hence, future research could evaluate the impact of an *oropharyngeal candidiasis* on the PiPPD and PiBOP and evaluate if the other findings of this retrospective analysis remain the same over a longer period of 5 or 10 years.

## 6 CONCLUSION

Within the limitations of this retrospective analysis, the authors conclude that adherence to peri‐implant maintenance visits is low and deserves attention. In those patients that comply with the annual maintenance schedule during the 3.5 years after implant surgery, a stable peri‐implant condition was maintained. As future consideration, the comparison of the clinical outcomes of patients participating in the maintenance program with those that did not, would make this observation even more meaningful.

## CONFLICT OF INTEREST

This retrospective analysis was self‐funded by the authors and therefore the authors can report that they have no conflict of interest with any commercial brand or entity mentioned in this report. This research received no specific grant from any funding agency in the public, commercial, or non‐profit sectors. This report was prepared as a part of the obligation of the first and second author to fulfill the requirements of the ACTA bachelor's program in Dentistry. Fridus August van der Weijden is the owner of the Clinic for Implantology Utrecht.

## Data Availability

Supplemental information that support the findings of this study, such as raw data, are available from the corresponding author upon reasonable request.
